# Development and Optimization of Rice and Teff Based Gluten-Free Mixes for Traditional Algerian Pancakes: Evaluation of Technological Properties, Nutritional Quality, and Sensory Attributes

**DOI:** 10.3390/foods15111867

**Published:** 2026-05-25

**Authors:** Awatif Fetouhi, Hayat Bourekoua, Radia Ayad, Fairouz Djeghim, Meryem Bouchrit, Amina Mosbah, Khawla Kerbab, Maria D’Elia, Luca Rastrelli, Soued Cherak

**Affiliations:** 1 Laboratoire de Génie Agro-Alimentaire (GENIAAL), Institut de la Nutrition de l’Alimentation et des Technologies Agro-Alimentaires (INATAA), Université Constantine 1 Frères Mentouri, Route de Ain El-Bey, Constantine 25000, Algeria; boucherit.meryam@gmail.com (M.B.); mosbahamina9@gmail.com (A.M.); 2 Equipe Formulation des Nouveaux Produits Agro-Alimentaires (FNPAA), Laboratoire de Nutrition et Technologie Alimentaire (LNTA), Institut de la Nutrition, de l’Alimentation et des Technologies Agro-Alimentaires (INATAA), Université Constantine 1 Frères Mentouri, Route de Ain El-Bey, Constantine 25000, Algeria; bourekoua.hayat@umc.edu.dz (H.B.); fairouze.djeghim@umc.edu.dz (F.D.); 3 Valorization of Natural Resources, Bioactive Molecules and Biological Analysis Unit, Department of Chemistry, Université Constantine 1 Frères Mentouri, Route de Ain El-Bey, Constantine 25000, Algeria; radia.ayad@yahoo.fr; 4 Laboratory of Phytochemistry and Pharmacology, Department of Chemistry, Faculty of Exact Sciences and Informatics, University Mohammed Seddik Benyahia of Jijel, Jijel 18000, Algeria; 5 Laboratoire de Génie Biologique Valorisation et Innovation des Produits Agroalimentaires, Institut ISTA-Ain M’Lila, Université Larbi Ben M’hidi Oum El-Bouaghi, Oum El-Bouaghi 04000, Algeria; kerbab.khawla@univ-oeb.dz; 6 Department of Pharmacy, University of Salerno, Via Giovanni Paolo II, 132, 84084 Fisciano (SA), Italy; mdelia@unisa.it; 7 Italy National Biodiversity Future Center (NBFC), 90133 Palermo, Italy; 8 Dipartimento di Scienze della Terra e del Mare, University of Palermo, 90123 Palermo, Italy; 9 Institut de la Nutrition, de l’Alimentation et des Technologies Agro-Alimentaires (INATAA), Université Constantine 1 Frères Mentouri, Route de Ain El-Bey, Constantine 25000, Algeria; cheraksouade@gmail.com

**Keywords:** traditional Algerian pancakes, teff, rice, mixture design, technological properties, nutritional quality, sensory attributes

## Abstract

Gluten-free fermented products remain technologically challenging due to the absence of gluten, which plays a key role in stabilizing batter structure and gas retention. This study proposes a mixture design-driven approach to develop gluten-free Algerian pancakes based on rice and teff formulations enriched with legumes and seeds, aiming to restore techno-functional properties while improving nutritional quality. Two formulations, a teff-based (TBF) and a rice-based (RBF) system, were optimized using a simplex centroid mixture design and evaluated in comparison with durum wheat pancakes. The results demonstrated that formulation strongly influenced batter rheology and final structure. The TBF system exhibited superior technological performance, with higher specific volume (1.77 cm^3^/g), lower density (0.56 g/cm^3^), and enhanced porosity, associated with improved protein and fiber content. In contrast, the RBF formulation showed higher antioxidant activity. The findings highlight the critical role of component interactions in modulating batter viscosity and foam stability, which directly affected pore development and product airiness. Both optimized formulations successfully reproduced the characteristic “light and airy” structure of traditional pancakes, achieving good sensory acceptability. Overall, this study demonstrates that mixture design can effectively guide the development of gluten-free fermented systems by linking composition, rheology, and structural properties, providing a strategy for improving the quality of traditional gluten-free foods.

## 1. Introduction

Celiac disease is an immune-related disorder triggered by the ingestion of prolamins from cereals such as wheat, barley, rye, and oats. In genetically predisposed individuals, these proteins induce inflammation of the intestinal mucosa, leading to villous atrophy, epithelial lymphocytosis, and impaired nutrient absorption [1]. Consequently, severe malabsorption of vitamins, minerals, proteins, carbohydrates, and lipids may occur [2], often resulting in clinical complications such as growth impairment, particularly in children [3]. Lifelong adherence to a gluten-free diet is therefore essential, although such diets must be carefully formulated to compensate for nutritional deficiencies associated with malabsorption.

Despite the growing availability of gluten-free products, many formulations are still based on refined starches, resulting in products with poor nutritional profiles, typically characterized by high fat and salt content but low protein and dietary fiber [4]. This contrasts with current dietary recommendations promoting fiber-rich foods and balanced lipid intake [5]. From a technological perspective, gluten plays a crucial role in forming a viscoelastic network that governs dough structure and gas retention during processing [6]. Its removal therefore represents a major challenge, particularly in fermented or aerated systems where batter rheology and foam stability are critical. Consequently, the development of gluten-free products with adequate structural and sensory properties remains a key challenge for food scientists [7,8,9].

To address these limitations, recent research has focused on the use of alternative gluten-free flours derived from cereals (e.g., rice, millet), pseudocereals (e.g., quinoa, amaranth), and legumes (e.g., chickpea, lupine), aiming to improve both nutritional value and techno-functional performance [10,11]. Among these, rice flour is widely used due to its hypoallergenic nature and neutral taste; however, it lacks sufficient protein and fiber to support optimal structure formation [12,13]. In contrast, teff and pseudocereals such as quinoa provide enhanced nutritional profiles and functional properties. Teff is particularly rich in dietary fiber and polyphenols, while quinoa contributes essential fatty acids and starch characteristics that can partially mimic wheat functionality, improving both rheology and sensory attributes [14,15,16,17].

Legume flours, including chickpea and lupine, further contribute to improving gluten-free systems by providing high-quality proteins and low glycemic index carbohydrates. Importantly, they also exhibit surface-active properties that promote foam stabilization, which is essential for the formation of aerated structures in leavened products [18,19,20]. This effect can be enhanced by the incorporation of functional oilseeds such as chia, flaxseed, and garden cress. These seeds are rich in bioactive compounds and mucilage, acting as natural hydrocolloids with high water-holding capacity. Their presence contributes to viscosity modulation, foam stability, and emulsification, all of which are critical parameters in gluten-free batter systems [21,22,23,24].

Among gluten-free foods, pancakes represent an interesting model system due to their fermented batter structure and their reliance on gas retention and pore formation to achieve the characteristic light and airy texture. In North African regions such as Algeria, traditional pancakes are prepared from semolina-based fermented batters, producing a distinctive product characterized by a porous surface and a soft internal structure [25]. Replicating this complex structure in gluten-free formulations remains particularly challenging due to the absence of gluten.

Although previous studies have explored gluten-free pancake formulations, most have focused primarily on consumer acceptability or simple ingredient substitutions [26,27]. There is still a lack of systematic approaches addressing the optimization of multi-component gluten-free systems combining cereals, pseudocereals, legumes, and seeds, as well as their impact on batter rheology and final product structure. In this context, mixture design represents a powerful statistical tool to investigate interactions between ingredients and to optimize formulation based on multiple quality parameters.

Therefore, the aim of this study was to develop and optimize gluten-free pancakes based on rice- and teff-based formulations enriched with legumes and seeds. A simplex centroid mixture design was applied to evaluate the influence of component interactions on technological, rheological, nutritional, and sensory properties. Additionally, a household survey was conducted to define the traditional preparation process, allowing the development of gluten-free formulations that reproduce the structural and sensory characteristics of traditional Algerian pancakes.

## 2. Materials and Methods

### 2.1. Chemicals and Reagents

1,1-Diphenyl-2-picrylhydrazyl (DPPH), 2,2′-azino-bis(3-ethylbenzothiazoline-6-sulfonic acid) (ABTS), ammonium molybdate, Folin–Ciocalteu reagent, ascorbic acid, gallic acid, quercetin, ferric chloride (FeCl_3_), potassium ferricyanide (K_3_Fe(CN)_6_), and trichloroacetic acid (TCA) were purchased from Sigma-Aldrich (Sigma-Aldrich, Steinheim, Germany). All other reagents and solvents were of analytical grade.

### 2.2. Raw Material

Teff (12.30% moisture, 2.39% ash, 12.5% protein, and 4.45% lipids), white millet (9.24% moisture, 1.33% ash, 12.22% protein, and 4.08% lipids), and quinoa (10.31% moisture, 2.29% ash, 13.39% protein, and 6.62% lipids) were provided by Natur Alim (Algiers, Algeria).

Rice (12.40% moisture, 0.35% ash, 8.96% protein, and 1.39% lipids), chickpea (8.17% moisture, 3.04% ash, 18.66% protein, and 5.67% lipids), and sweet lupine grains (7.93% moisture, 3.56% ash, 33.87% protein, and 12% lipids), as well as chia seeds (6.14% moisture, 3.98% ash, 16.85% protein, and 31% lipids), garden cress seeds (5.48% moisture, 4.26% ash, 21.25% protein, and 27.73% lipids), and flax seeds (6.40% moisture, 3.44% ash, 19.58% protein, and 38.88% lipids), were purchased from a local market in Constantine, Algeria.

Baker’s yeast, gluten-free baking powder, sunflower oil, and salt were also obtained locally.

Durum wheat semolina (11.08% moisture, 1.16% ash, 12.08% protein, and 1.16% lipids), used as the control, was purchased from the same market.

The chemical composition of teff, white millet, and quinoa was obtained directly from the product manufacturer. For rice, chickpea, sweet lupine, chia, flax, garden cress seeds, and durum wheat semolina, the composition was derived from product labels. Moisture and ash contents of all raw materials were determined experimentally.

### 2.3. Material Preparation

All raw materials were obtained in grain form. Garden cress, chia, flax seeds, rice, and sweet lupine grains were manually sorted to remove impurities and damaged kernels. Teff, millet, and quinoa were purchased pre-cleaned.

Sweet lupine grains were washed several times with water to remove dust and impurities, then air-dried. To enhance their foaming and stabilizing properties, the dried grains were subjected to heat treatment at 80 °C for 5 min, according to Pozani et al. [18].

Garden cress seeds were roasted at 120 °C for 7 min to reduce their characteristic pungent flavor while preserving their mucilage-forming capacity.

These thermal treatments were conducted using a Memmert oven (Memmert GmbH + Co. KG, Aeussere Rittersbacher Strasse 38, D-91126 Schwabach, FRG-Germany).

All prepared grains were ground using a Brandmann blade grinder (700 W, 28,000 rpm, Clatronic International Group, Kempen, Germany) and sieved through 500 and 200 μm meshes to obtain semolina with particle size between 200 and 500 μm, suitable for traditional pancake preparation.

The obtained semolina was stored in synthetic fiber bags under cool and dry conditions until use.

### 2.4. Gluten-Free Pancakes Formulation

**Figure 1:**
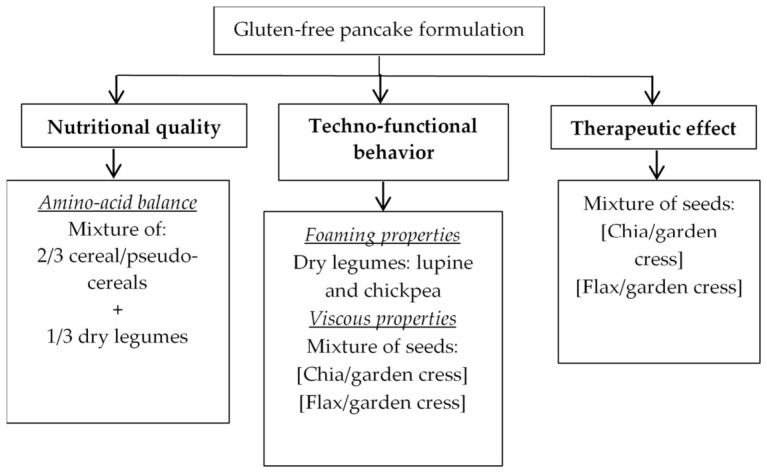
Principle of gluten-free pancake formulations.

The batter of traditional durum wheat pancakes was considered a viscous foam system, in which the viscoelastic properties of gluten contribute to gas retention and the formation of the characteristic honeycomb structure during baking. Based on this hypothesis, gluten-free formulations were designed to reproduce similar rheological and structural behavior while improving nutritional quality (Figure 1). The nutritional criterion (2/3 cereals–1/3 legumes) was applied as a complementary framework to validate protein quality through amino acid balance, consistent with established principles of cereal-legume complementation [28,29,30].

Two gluten-free mixes were developed, each composed of a combination of cereals/pseudocereals, dry legumes, and seeds. The first formulation (TBF) included teff, millet, and quinoa as the cereal/pseudocereal base, chickpea as the legume source, and a mixture of flax and garden cress seeds. The second formulation (RBF) consisted of rice, millet, and quinoa, supplemented with lupine and a mixture of chia and garden cress seeds (Table 1). The proportions of cereal/pseudocereal mixtures and seed combinations were determined through preliminary trials based on technological and sensory evaluation. Chia and flax seeds were included to mitigate the pungent flavor of garden cress while maintaining its thickening properties. Millet was incorporated to improve both color and sensory acceptability of the formulations.

### 2.5. Mixture Design

To evaluate the effect of formulation components on pancake quality and to optimize their relative proportions, a simplex centroid mixture design was applied. Three independent variables were considered: X_1_ (cereal/pseudocereal mixture), X_2_ (legume fraction), and X_3_ (seed mixture), under the mixture constraint (Equation (1)):

X_1_ + X_2_ + X_3_ = 100 (1)

Two separate mixture designs were developed for teff-based (TBF) and rice-based (RBF) formulations.

The experimental data were fitted using a canonical Scheffé polynomial model for mixture systems (Equation (2)):

Y = β_1_X_1_ + β_2_X_2_ + β_3_X_3_ + β_12_X_1_X_2_ + β_13_X_1_X_3_ + β_23_X_2_X_3_ + β_123_X_1_X_2_X_3_ (2)

where Y represents the predicted response, β_i_ the linear coefficients, β_ij_ the binary interaction coefficients, and β_ijk_ the ternary interaction coefficient.

The selected response variables were chosen to reflect the technological and structural properties of fermented pancakes: specific volume (SV, cm^3^/g) (Y_1_), porosity (Y_2_), color expressed as L* (Y_3_), and batter rheology expressed as kinematic viscosity (cSt) (Y_4_).

The optimization objective was to obtain gluten-free pancakes with technological properties comparable to those of durum wheat-based pancakes.

The ranges of mixture components were defined based on preliminary trials considering both technological (specific volume and porosity) and sensory attributes (color and taste). For TBF, the cereal/pseudocereal fraction ranged from 70 to 79% (*w/w*), legumes from 20 to 29% (*w/w*), and seeds from 1 to 10% (*w/w*). For RBF, the cereal/pseudocereal fraction ranged from 75 to 84% (w/w), legumes from 15 to 24% (*w/w*), and seeds from 1 to 10% (*w/w*) (Table 2).

The desirability function approach was applied to simultaneously optimize the multiple response variables. Each response was transformed into an individual desirability function (d_i_) ranging from 0 (undesirable) to 1 (optimal), and the overall desirability was calculated as the geometric mean of individual desirabilities.

### 2.6. Traditional Algerian Pancakes Preparation

#### 2.6.1. Household Survey

Traditional Algerian pancakes remain poorly characterized from a scientific and technological perspective. To establish a standardized preparation protocol, a household survey was conducted in two cities in eastern Algeria (Bordj Bou Arréridj and Souk Ahras), where this product is widely consumed.

A total of 200 women aged between 19 and 87 years participated in the survey. The selection was based on their direct involvement in traditional pancake preparation, which is predominantly performed by women in household settings.

Data were collected through structured interviews conducted in the local language to ensure accuracy and consistency. Participants who were not familiar with the traditional preparation process were excluded. Verbal informed consent was obtained from all participants, who voluntarily agreed to take part in the study.

The questionnaire was developed and validated through a preliminary pre-survey involving a subset of participants. It was structured into three sections: (i) demographic information, (ii) local denominations of traditional pancakes, and (iii) detailed preparation practices, including ingredients, proportions, type of semolina, and processing steps.

#### 2.6.2. Preparation of Gluten-Free Traditional Pancakes

To ensure reproducibility under controlled conditions, the traditional preparation process was adapted by replacing manual kneading steps with a standardized mechanical mixing procedure. Key parameters, including water content, mixing time, fermentation duration, homogenization time, and yeast concentration, were defined based on preliminary trials (Figure S1, Supplementary Materials).

For each formulation, 100 g of semolina (durum wheat for control or gluten-free mixtures defined by the mixture design), 3 g of salt, 3 g of baker’s yeast, 3 g of baking powder, and 10 mL of sunflower oil were mixed with an appropriate amount of distilled water. Initial mixing was performed manually for 2 min, followed by mechanical mixing using a Bosch electric hand mixer (MFQ3030, Stuttgart, Germany) for 20 min to obtain a homogeneous liquid batter.

The batter was then fermented at 40 °C for 45 min using a Memmert oven (Memmert GmbH + Co. KG Aeussere Rittersbacher Strasse 38 D-91126 Schwabach FRG-Germany). Following fermentation, the system exhibited a foam-like structure due to CO_2_ production. After fermentation, a controlled homogenization step was applied to regulate gas distribution and stabilize the foam structure. This was achieved by manual stirring with a ladle for 30 s every 2 min over a 10 min period.

This step was found to be critical for achieving a uniform porous structure and the characteristic light and airy texture of the final product.

A volume of 10 mL of fermented batter was poured onto a preheated ceramic plate (230–250 °C) and baked on one side for 2–3 min. To prevent sticking, a thin layer of oil or egg wash was applied to the plate surface before each baking cycle.

The complete preparation process is illustrated in Figure S1 (Supplementary Materials).

### 2.7. Quality Assessment of Liquid Batter and Gluten-Free Pancakes

The quality of liquid batter and the resulting pancakes was evaluated through rheological and structural analysis, which are key parameters governing the formation of the characteristic porous structure of traditional Algerian pancakes.

The rheological behavior of the liquid batter was assessed by measuring kinematic viscosity (ν), as viscosity plays a critical role in gas retention and bubble stability during fermentation and baking. Measurements were performed using an ISO 2431 [31] flow cup equipped with a 6.0 mm orifice (Selecta, Barcelona, Spain), due to its suitability for low-viscosity batter systems. The batter temperature was controlled at 21 °C prior to testing. Efflux time was recorded and converted into kinematic viscosity (cSt) using the Elcometer ElcoCalc™ application (version 1.9.0, Elcometer, Manchester, England). The analysis was conducted in three replicates.

The technological quality of pancakes was evaluated through specific volume, alveolar structure, and color measurements. According to the household survey results, a successful Algerian pancake is characterized by a light, thin texture, a highly perforated surface, and a light yellowish color.

Specific volume (cm^3^/g), which reflects the degree of aeration and product lightness, was determined using the rapeseed displacement method according to AACC method 10.05 [32]. Pancakes were cut, stacked, and wrapped prior to measurement, and all analyses were performed in triplicate.

The alveolar structure was analyzed using digital image processing according to the method described by Djeghim et al. [33]. Pancakes were photographed under controlled lighting conditions in a closed polystyrene box equipped with LED illumination. Images were acquired using a smartphone camera (Galaxy S10), saved in TIFF format, and processed using ImageJ software (version 1.43u, NIH, Bethesda, MD, USA). After conversion to 8-bit grayscale, thresholding was applied to distinguish pores from the solid matrix [10]. The number of cells per pancake and the average pore size were quantified. The values were the means of three replicates.

Color measurements were performed according to the method described by Djeghim et al. [33], using the Color Grab application (version 3.6.1, Loomatix Ltd., Munich, Germany) under standardized lighting conditions. The CIE Lab* color space was used, where L* represents lightness, a* the green–red axis, and b* the blue–yellow axis. Measurements were taken at five different points on the honeycomb surface of each sample. Three replicates were conducted.

### 2.8. Antioxidant Properties

The antioxidant properties of gluten-free pancakes and durum wheat control samples were evaluated through total phenolic content (TPC), total flavonoid content (TFC), total antioxidant capacity (TAC), and radical scavenging assays (DPPH and ABTS), as well as reducing power.

Extraction was performed according to the method described by Ayad et al. [34]. Briefly, 1 g of dried and powdered pancake sample was sonicated in 10 mL of 80% ethanol at 40 °C for 1 h using an ultrasonic bath (Ultrasons-H, 50/60 Hz, 720 W, Barcelona, Spain). The extracts were cooled and filtered through Whatman No. 1 filter paper prior to analysis.

Total phenolic content (TPC) was determined using the Folin–Ciocalteu method described by Singleton and Rossi [35], with slight modifications. An aliquot of 0.30 mL extract was mixed with 1.20 mL of diluted Folin–Ciocalteu reagent (1:10), followed by the addition of 1.50 mL of 7.5% Na_2_CO_3_. After incubation for 2 h in the dark at room temperature, absorbance was measured at 765 nm. Results were expressed as mg gallic acid equivalents per gram of dry weight (mg GAE/g d.w).

Total flavonoid content (TFC) was determined according to Djeridane et al. [36]. Briefly, 1 mL of extract was mixed with 1 mL of 2% AlCl_3_ solution, and absorbance was measured at 430 nm after 10 min. Results were expressed as mg quercetin equivalents per gram of dry weight (mg QE/g d.w).

Total antioxidant capacity (TAC) was evaluated using the phosphomolybdate method described by Prieto et al. [37]. A volume of 0.3 mL extract was mixed with 3 mL reagent solution (0.6 M sulfuric acid, 28 mM sodium phosphate, and 4 mM ammonium molybdate) and incubated at 95 °C for 90 min. Absorbance was measured at 695 nm, and results were expressed as mg ascorbic acid equivalents per gram dry weight (mg AAE/g d.w).

The free radical scavenging activity against DPPH was determined according to Ismail et al. [38], with modifications. Extracts were prepared in methanol (1 mg/mL) and diluted to different concentrations. A volume of 2 mL extract was mixed with 2 mL of 0.2 M DPPH ethanolic solution and incubated in the dark for 30 min. Absorbance was measured at 517 nm, and results were expressed as IC_50_ (mg dw/mL).

Reducing power was determined according to Oyaizu [39], as reported by Boonchum et al. [40] and Luqman et al. [41]. Extracts were mixed with phosphate buffer (0.2 M, pH 6.6) and potassium ferricyanide (1%, *w/v*), incubated at 50 °C for 20 min, and then treated with trichloroacetic acid (10%). After centrifugation, the supernatant was mixed with distilled water and FeCl_3_ (0.1%, *w/v*), and absorbance was measured at 700 nm. Results were expressed as A_0_._5_ (mg dw/mL), with gallic acid used as a standard.

ABTS radical scavenging activity was evaluated according to Re et al. [42]. The ABTS●^+^ radical solution was prepared by reacting 7 mM ABTS with 2.45 mM potassium persulfate and incubating the mixture in the dark for 12–16 h. The working solution was diluted to an absorbance of 0.70 ± 0.02 at 734 nm. Then, 2 mL of ABTS●^+^ solution was mixed with 1 mL of extract, incubated for 10 min in the dark, and the absorbance was measured at 734 nm. Results were expressed as IC_50_ (mg dw/mL).

All analyses were performed in triplicate.

### 2.9. Proximate Composition Analysis

The proximate composition of the optimized gluten-free formulations and durum wheat semolina (control) was determined. Moisture, protein, lipid, and ash contents were analyzed according to AOAC methods 926.12, 960.52, 996.01, and 942.05, respectively [43].

Crude fiber content was determined using the Weende method [44]. Carbohydrate content was calculated by difference, subtracting the sum of protein, lipid, ash, and crude fiber from 100 g of dry matter.

The energy value (kcal/100 g) was calculated using Atwater conversion factors: 9 kcal/g for lipids and 4 kcal/g for proteins and carbohydrates, according to Costantini et al. [45].

All analyses were performed in triplicate.

### 2.10. Sensorial Analysis

Sensory evaluation of gluten-free pancakes based on optimized formulations and durum wheat control samples was conducted using a trained panel of 65 assessors familiar with sensory evaluation procedures. Prior to the test, participants were informed about the study and provided their consent in accordance with the ethical guidelines of the INATAA Institute (Constantine 1 Frères Mentouri University).

Each assessor evaluated three samples: teff-based formulation (TBF), rice-based formulation (RBF), and durum wheat control. Pancakes were prepared on the day of evaluation, served warm, and accompanied by a standardized topping (vegetable butter and granulated sugar).

A nine-point hedonic scale (1 = dislike extremely; 9 = like extremely) was used to evaluate sensory attributes, including porosity, taste, aroma, texture, color, airiness and overall acceptability [46].

In addition, overall preference among samples was assessed using a ranking test according to the method described by Watts et al. [47].

### 2.11. Statistical Analysis

Results were expressed as mean ± standard deviation. The mixture design, regression modeling, and contour plots were generated using Minitab 2019 software (Minitab LLC, USA). Model adequacy was evaluated based on the coefficient of determination (R^2^), analysis of variance (ANOVA, F-test), and residual diagnostics. The desirability function approach was applied for multi-response optimization using Minitab Release 19 (Minitab Inc., State College, PA, USA). One-way analysis of variance (ANOVA), followed by Tukey’s post hoc test, was used to determine significant differences among samples for rheological, technological, antioxidant, sensory, and compositional parameters, with a significance level set at *p* < 0.05. Correlation analysis between variables was performed using XLSTAT 2009 software (version 2009.1.01, Addinsoft).

## 3. Results and Discussion

### 3.1. Survey Outcomes: Traditional Ingredients and Preparation Process of Algerian Pancakes

The demographic distribution of the surveyed population showed that 20% of participants were aged between 19 and 30 years, 44% between 30 and 50 years, and 36% between 50 and 87 years. All respondents reported preparing traditional pancakes at home, confirming the strong cultural relevance and widespread domestic production of this product.

According to the survey, traditional Algerian pancakes are known under different local denominations, including Korsa, Ghraif, Thighrifine, and Baghrir, reflecting regional variations in naming rather than formulation. Despite this diversity, all participants (100%) consistently reported the use of durum wheat semolina, salt, water, and baker’s yeast as the fundamental ingredients. Additional ingredients such as eggs and vegetable oil were occasionally incorporated to enhance alveolar structure and texture, respectively.

The traditional preparation process involves sequential hydration and manual kneading steps to progressively transform semolina into a liquid batter suitable for fermentation. Initially, semolina, salt, and yeast are mixed with warm water to obtain a semi-solid dough, followed by manual kneading for approximately 15 min. Subsequently, additional water is gradually incorporated with continuous kneading until a homogeneous liquid batter is obtained. This batter is then fermented for 45–60 min at ambient temperature prior to baking. Cooking is traditionally performed on a heated clay plate (Tdjine), resulting in pancakes characterized by a porous honeycomb structure and a soft internal texture.

Interestingly, 76% of respondents strictly followed this traditional multi-step process, whereas 24% adopted a simplified approach by replacing the two kneading steps with a single mixing step, in which all ingredients are hydrated simultaneously to obtain a liquid batter. This variation suggests a degree of process flexibility while maintaining the essential structural characteristics of the final product.

**Figure 2:**
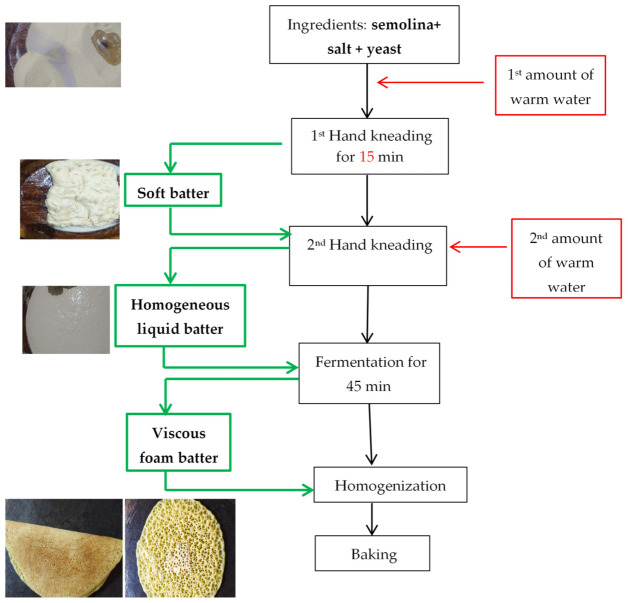
Traditional preparation process of Algerian pancakes derived from household survey data, highlighting sequential hydration, kneading, fermentation, and baking steps.

The preparation diagram derived from the survey is presented in Figure 2. This traditional process highlights the importance of progressive hydration, mechanical development, and controlled fermentation in determining batter rheology and, ultimately, the formation of the characteristic porous structure.

### 3.2. Mixture Design Analysis and Model Validation

The regression coefficients of the linear mixture models describing the effect of formulation components (X_1_: cereal/pseudocereal mixture, X_2_: legume fraction, and X_3_: seed mixture) on technological and rheological responses are presented in Table 3.

Overall, the results indicate that all three components significantly influenced the investigated responses in most cases (*p* < 0.05), confirming the suitability of the mixture design approach to describe the behavior of the system. In contrast, interaction terms were found to be non-significant, suggesting that the main effects of individual components dominate the response variability within the selected formulation ranges.

As previously reported by Bourekoua et al. [48], the sign and magnitude of regression coefficients provide insight into the direction and intensity of each component’s effect. Positive coefficients indicate that increasing the proportion of a component enhances the response, whereas negative coefficients indicate an inverse relationship.

Among the studied variables, the seed fraction (X_3_) emerged as the most influential factor affecting pancake quality. A strong positive effect of X_3_ on lightness was observed in both formulations (coefficients of 21.43 for TBF and 35.96 for RBF), indicating that seed incorporation contributes to brighter product appearance. However, this effect was accompanied by a marked negative impact on porosity, particularly in the TBF system (−1153), suggesting that excessive seed addition leads to structure densification and reduced pore formation.

This behavior highlights a critical formulation trade-off: while seeds improve optical properties and potentially nutritional value, their excessive incorporation compromises the development of the alveolar network. Therefore, careful optimization of seed content is required to balance visual and structural attributes.

Differences between the two matrices were particularly evident in porosity behavior. In the RBF system, cereals and legumes contributed positively to porosity, promoting a more aerated structure. In contrast, in the TBF system, both cereal and seed fractions exhibited negative contributions, indicating a stronger tendency toward structure densification. These results suggest that the structural response of the batter is highly matrix-dependent.

The analysis of viscosity further confirmed this divergence between teff- and rice-based systems. In the TBF formulation, increasing the proportion of any component (X_1_, X_2_, or X_3_) resulted in a decrease in viscosity, with seeds exerting the strongest thinning effect (−41.19). This behavior suggests disruption of the teff-based starch–protein network, leading to a more fluid batter.

Conversely, in the RBF formulation, while cereals and legumes reduced viscosity, seeds (X_3_) produced a significant increase (39.18), indicating a thickening effect. This opposite behavior suggests that seed components, likely due to their fiber and lipid content, interact differently with rice-based matrices, acting as structuring agents rather than network disruptors.

These contrasting rheological responses demonstrate that teff and rice cannot be considered functionally equivalent in gluten-free systems and require formulation-specific optimization strategies.

Model adequacy analysis showed an excellent fit for the TBF formulation, with coefficients of determination (R^2^) consistently exceeding 99%, indicating that the variation in responses was almost entirely explained by mixture composition. For the RBF formulation, the models also showed high explanatory power (R^2^ generally > 96%), except for specific volume (SV), which exhibited a lower R^2^ (79.54%) and a non-significant effect (*p* > 0.05).

This suggests that, in the rice-based system, factors beyond formulation composition—such as processing conditions or batter aeration dynamics—may play a more dominant role in determining specific volume.

Overall, the results confirm that the developed mixture models adequately describe the relationships between formulation variables and quality attributes, supporting their use for subsequent optimization.

### 3.3. Effect of Mixture Composition on Batter Rheology and Pancake Technological Properties

The effects of mixture composition on the rheological behavior of liquid batter and the technological properties of gluten-free pancakes are presented in Table 4 and Table 5 for teff-based (TBF) and rice-based (RBF) formulations, respectively.

Analysis of variance (ANOVA) revealed significant differences among the seven experimental trials (*p* < 0.05), confirming that the proportions of cereal/pseudocereal mixture (X_1_), legume fraction (X_2_), and seed mixture (X_3_) strongly influence both batter rheology and final product characteristics.

**Figure 3:**
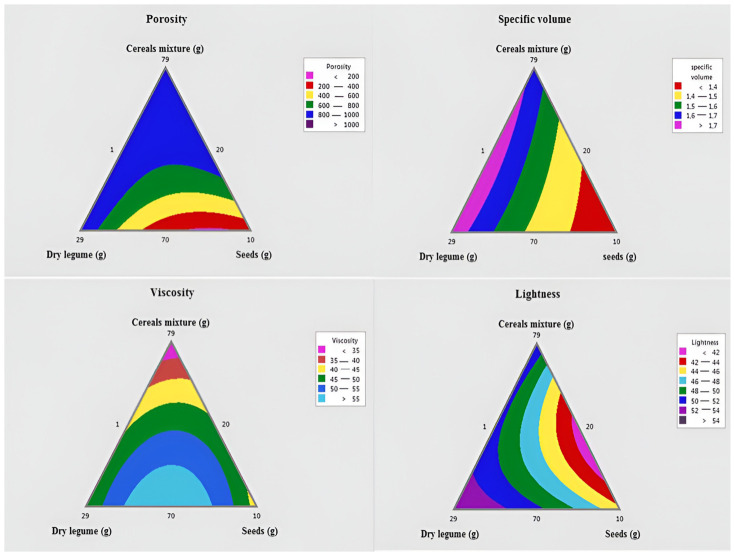
Triangular contour plots of specific volume (SV), porosity, lightness (L), and viscosity for teff-based formulations.

**Figure 4:**
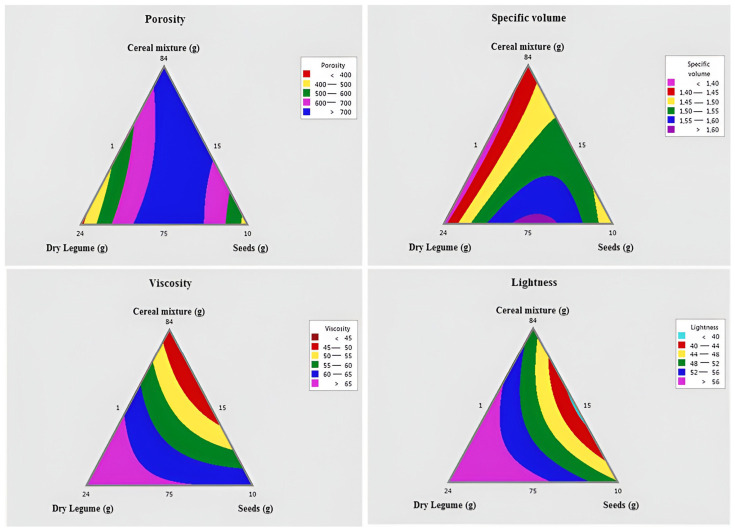
Triangular contour plots of specific volume (SV), porosity, lightness (L), and viscosity for rice-based formulations.

**Figure 5:**
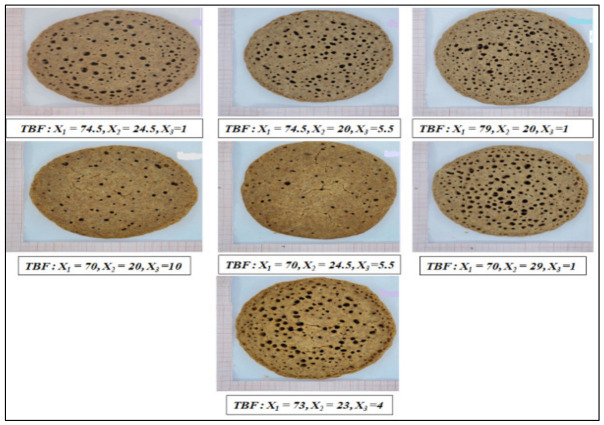
TBF-based pancake samples corresponding to the seven trials of the experimental design.

**Figure 6:**
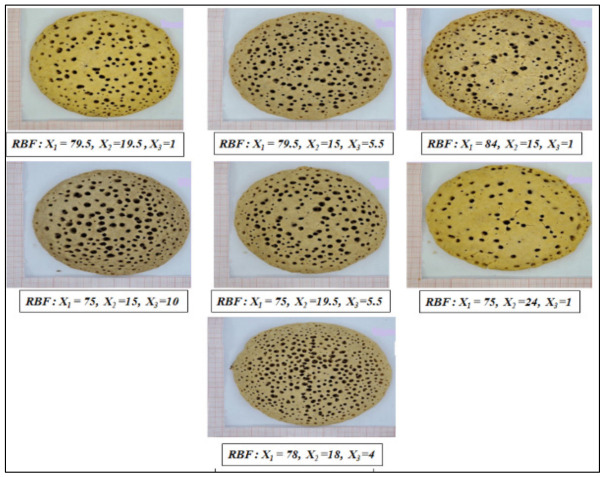
RBF-based pancake samples corresponding to the seven trials of the experimental design.

The triangular contour plots (Figure 3 and Figure 4) provide a comprehensive visualization of the response surfaces, highlighting the combined effects of mixture components on specific volume (SV), porosity, lightness (L*), and viscosity. In addition, representative images of pancakes obtained from each experimental trial are provided in Figure 5 and Figure 6 for TBF and RBF, respectively.

#### 3.3.1. Teff-Based Formulation (TBF)

As shown in Table 4, all measured quality attributes differed significantly among the seven formulations (*p* < 0.05), confirming the strong influence of mixture composition on both batter rheology and pancake structure.

In the TBF system (Table 4), specific volume values ranged from 1.35 to 1.78 cm^3^/g. The highest values were observed in formulations with minimal seed content and balanced cereal–legume proportions (F1 and F6: 1.78 and 1.77 cm^3^/g, respectively), which also exhibited the greatest porosity (936 and 1010, respectively). This confirms that low seed incorporation promotes optimal aeration and alveolar development. In contrast, formulations with higher seed fractions (F4 and F5) showed the lowest specific volume (1.35 and 1.48 cm^3^/g, respectively) and porosity (246.5 and 224.5, respectively), consistent with regression analysis identifying seeds as a major factor in structural densification. Porosity thus proved highly sensitive to formulation changes, with reduced pore formation accompanying increased seed levels.

Lightness (L*) values were positively influenced by seed incorporation, with maximum values recorded in F6 (L* = 54.26), supporting the role of seeds in enhancing optical properties. However, this improvement in visual quality occurred at the expense of structural integrity. The darkest sample was F2 (L* = 40.96), suggesting that intermediate seed levels combined with teff-based matrices negatively affect optical properties. Overall, the results highlight a trade-off: while seeds enhance lightness, they compromise aeration and porosity.

Batter viscosity ranged from 30.60 to 58.64 cSt. The highest viscosity (F5: 58.64 cSt) coincided with low specific volume and porosity, indicating that excessive batter thickness limits gas expansion during cooking. Conversely, the lowest viscosity (F3: 30.60 cSt) was associated with higher aeration (SV = 1.67 cm^3^/g; porosity = 795), suggesting that reduced viscosity facilitates bubble expansion. These findings emphasize that optimal batter consistency is required to balance gas retention and expansion.

Overall, the data indicate that high specific volume and porosity can be achieved under two compositional conditions: either high cereal content with low legume levels, or lower cereal content compensated by higher legume incorporation. In both cases, maintaining a low seed fraction remains critical to preserving structural integrity, while viscosity further modulates the balance between gas retention and expansion.

Contour plot analysis (Figure 3) revealed that optimal specific volume (>1.6 cm^3^/g) is achieved in regions characterized by high cereal and legume contents combined with low seed levels. Similarly, porosity exhibited a broad optimal region (>800), expanding toward higher cereal and legume proportions, while sharply decreasing at high seed concentrations.

The optimal lightness region (L* > 52) (Figure 3) was located at high legume content and low cereal and seed levels. This observation is consistent with previous findings reported by Ferradji et al. [11], where the yellowish color of legumes contributed positively to lightness, whereas the darker pigments of teff and seeds reduced brightness [15,16,24].

The viscosity contour plot (Figure 3) indicated that maximum values occur at low cereal levels combined with high seed and legume contents. However, the overlap of response surfaces identified an optimal viscosity range (35–50 cSt) that enables both gas retention and expansion.

This finding aligns with the mechanism proposed by Sumnu and Sahin [49], which states that optimal volume development requires a balance between batter stability and deformability. Excessively viscous systems hinder expansion, whereas overly fluid systems fail to retain gas.

Overall, the TBF system is highly sensitive to seed incorporation and requires precise control of formulation to balance structural, rheological, and optical properties.

#### 3.3.2. Rice-Based Formulation (RBF)

Analysis of variance (Table 5) revealed statistically significant differences among the seven formulations for all evaluated responses (*p* < 0.05), confirming the strong influence of mixture composition on both rheological and technological properties. 

In the RBF system (Table 5), specific volume (SV) values ranged from 1.38 to 1.63 cm^3^/g, showing a narrower variation compared to TBF. The highest value was observed for formulation F5 (1.63 cm^3^/g), characterized by balanced proportions of cereals (75%), legumes (19.5%), and seeds (5.5%). In contrast, the lowest SV values (1.38–1.39 cm^3^/g) were obtained for F6 and F1, respectively, both with minimal seed content (1%), regardless of cereal–legume proportions. These results indicate that, unlike the TBF system, moderate seed incorporation contributes positively to volume development, suggesting a structuring role within the rice-based matrix. This behavior highlights a synergistic interaction between mixture components, where balanced proportions are required to optimize expansion.

Porosity values ranged from 377.5 to 770, with higher values generally associated with moderate-to-high seed incorporation (F7, F3, F5, F2). Conversely, the lowest porosity (F6) was linked to low seed content and high legume proportion, indicating that excessive legume levels combined with insufficient structuring agents may limit pore formation. Unlike TBF, the negative impact of seeds on porosity was less pronounced, suggesting a different interaction mechanism between seeds and the rice-based matrix.

Lightness (L*) values were generally higher in RBF than in TBF, confirming the inherent color properties of rice-based systems. The highest lightness was recorded for F6 (L = 59.80), corresponding to low seed content and high legume levels, while the lowest value (L* = 38.65, F2) was associated with increased seed incorporation. This behavior is consistent with previous findings, where seed flours, particularly chia, contribute to darker product appearance due to phenolic compounds and intrinsic pigments [47,48]. Similarly, Steffolani et al. [49] reported a decrease in lightness with increasing seed content in gluten-free baked products, supporting the present observations.

Viscosity values in RBF ranged from 45.58 to 70.19 cSt. The highest viscosity (F6: 70.19 cSt) was observed in formulations with high legume content and minimal seed incorporation, whereas the lowest viscosity (F2: 45.58 cSt) corresponded to higher seed levels.

Notably, formulations with similar viscosity values (F1, F4, F5, F6, F7) exhibited markedly different technological properties, indicating that viscosity alone does not fully determine final product quality. Instead, foam stability and gas retention capacity appear to play a critical role, supporting the hypothesis that both rheological and interfacial properties govern structure formation.

Contour plot analysis (Figure 4) revealed that optimal SV values (>1.5 cm^3^/g) are achieved in regions characterized by moderate-to-high cereal content, moderate legume levels, and seed proportions close to their upper range. This contrasts with the TBF system, where low seed levels were required to maximize volume.

The optimal porosity region (>700) extended across a wide range of cereal compositions, provided that seed levels remained moderate to high and legume content was controlled.

The lightness contour plot indicated that maximum values were achieved at low seed content combined with high legume levels, whereas increasing seed incorporation led to darker products.

Finally, viscosity contour plots showed that high viscosity regions correspond to low SV and porosity, whereas lower viscosity zones favor expansion and aeration. This inverse relationship confirms that, in rice-based systems, excessive batter thickening hinders gas expansion, while moderate viscosity promotes optimal structure formation.

Overall, the RBF system exhibits a fundamentally different response to seed incorporation compared to TBF, where seeds act as structuring agents rather than network disruptors. This highlights the matrix-dependent functionality of ingredients and underscores the need for formulation-specific optimization strategies.

In TBF formulations, the higher intrinsic fiber and phenolic content of teff flour already contribute to elevated batter viscosity and reduced gas cell expansion [16]. When seeds are added, their mucilage further thickens the batter, exacerbating densification and limiting alveolar development during fermentation and impeding the migration and expansion of CO_2_, during cooking, leading to a lack of aeration [49]. This explains the negative correlation between seed fraction and porosity observed in TBF pancakes. Similar mechanisms have been reported in gluten-free systems where hydrocolloid-rich ingredients restrict bubble growth and reduce crumb openness by their highest gum viscosity [24,50,51].

In contrast, rice flour has lower fiber and protein content and forms weaker viscoelastic networks. Here, moderate seed incorporation improved porosity by providing mucilage and soluble fibers that increased viscosity, enhanced foam stability and gas retention [48]. Seeds acted as structuring agents, compensating for the weaker rice matrix. This synergistic effect explains why porosity increased with moderate seed levels in RBF. Similar findings were reported by Iglesias-Puig and Haros [52], who showed that chia flour improved gas cell stability in rice-based gluten-free breads.

As highlighted by Sciarini et al. [50], the impact of functional additives is strongly dependent on the characteristics of the raw material, the type and level of additive incorporated, and the availability of water. Consequently, predicting the precise effect of seed incorporation across different cereal–legume matrices is complex, since the same ingredient may act either as a structuring agent or as a network disruptor depending on the formulation.

### 3.4. Mixture Design Optimization

The desirability function approach was applied to optimize the quality attributes of gluten-free pancakes by simultaneously maximizing specific volume (SV), porosity, and lightness, while maintaining batter viscosity within an optimal range. Individual and composite desirability values for the optimized formulations are presented in Table 6.

As previously reported by Bourekoua et al. [48], optimal conditions are achieved when desirability values approach unity, indicating that all targeted responses are simultaneously satisfied.

The optimized teff-based formulation (TBF) was composed of 70.1% cereal/pseudocereal mixture (X_1_), 28.9% legume fraction (X_2_), and 1% seed mixture (X_3_), yielding a composite desirability of 0.989. All individual desirability values were close to 1 (0.99 for SV and viscosity; 0.98 for porosity and lightness) (Table 6), indicating excellent optimization of the system.

For the rice-based formulation (RBF), the optimal composition consisted of 75.54% cereal/pseudocereal mixture, 18.99% legume fraction, and 5.47% seed mixture, with a composite desirability of 0.91. Individual desirability values ranged from 0.80 (porosity) to 0.99 (viscosity), indicating satisfactory but less optimal performance compared to TBF.

Under optimal conditions, the predicted values for TBF were 1.77 cm^3^/g (SV), 1003 (porosity), 54.08 (L*), and 44.91 cSt (viscosity), while the corresponding observed values were 1.77 ± 0.06 cm^3^/g, 977 ± 3.45, 54.76 ± 2.6, and 44.94 ± 3.53 cSt, respectively (Table 6).

For RBF, predicted values were 1.60 cm^3^/g (SV), 756.05 (porosity), 55.66 (L*), and 64.20 cSt (viscosity), whereas observed values were 1.61 ± 0.01 cm^3^/g, 782.33 ± 9.61, 56.34 ± 0.34, and 61.41 ± 2.12 cSt, respectively (Table 6).

No significant differences were observed between predicted and experimental values for either formulation (*p* > 0.05), confirming the reliability and predictive accuracy of the developed models.

According to Djeghim et al. [53], the absence of significant differences between predicted and observed values demonstrates the validity of the statistical model. Therefore, the present results confirm that the mixture design approach is effective for optimizing gluten-free pancake formulations.

Overall, the TBF system exhibited superior optimization performance compared to RBF, suggesting that teff-based matrices are more responsive to formulation tuning and allow better control of structural and rheological properties.

The superior optimization performance of the TBF system compared to RBF can be explained by the intrinsic properties of teff flour. Teff, being rich in dietary fiber, proteins, and phenolic compounds, provides a stronger viscoelastic framework than rice flour, enabling formulation variables such as seed fraction and cereal–legume ratio to exert more predictable effects on porosity and viscosity. Alemneh et al. [54] highlighted that teff’s superior water absorption capacity plays a crucial role in gluten-free formulations, enhancing processing behavior and product quality. This functionality is linked to the protein matrix’s water-holding capacity, which allows water to be absorbed and retained through bound, hydrodynamic, capillary, and physically entrapped mechanisms [55]. In teff systems, this high hydration potential improves foam stability, gas retention, and alveolar development [56], whereas rice flour’s weaker networks result in less consistent responses that rely more heavily on stabilizing agents (viscosity and interfacial stabilization) [57]. These findings align with Sciarini et al. [50], who emphasized that the impact of additives is highly dependent on the raw material.

### 3.5. Characteristics of Optimized Gluten-Free Pancakes

#### 3.5.1. Proximate Composition and Calorific Value

The proximate composition and calorific value of optimized gluten-free formulations compared with the durum wheat control (CDW) are presented in Table 7. Statistical analysis revealed significant differences (*p* < 0.05) for all measured parameters, highlighting the strong impact of ingredient selection on the nutritional profile of the final products.

Moisture content ranged from 9.54% (TBF) to 11.07% (CDW), with gluten-free formulations exhibiting lower values than the control. These differences are likely related to the distinct hygroscopic behavior of the raw materials and their starch composition, which plays a key role in water-binding capacity [58]. All samples showed moisture levels below 14%, ensuring good microbiological stability and extended shelf life [59,60].

Protein content was significantly higher in gluten-free formulations compared to CDW, particularly for TBF (18.59%), followed by RBF (13.41%) and CDW (12.08%). These values fall within or above the upper range reported for gluten-free cereal- and pseudocereal-based products, where protein contents typically range from approximately 5.7% to 25.3% depending on the raw materials and formulation strategy [61].

In particular, pseudocereals such as amaranth and quinoa generally contain around 17–19% protein, confirming that the TBF formulation is positioned at the higher end of nutritionally enriched gluten-free products.

This enrichment reflects the combined contribution of pseudocereals, legumes, and seeds, which are known to improve both protein quantity and quality in gluten-free systems [61,62,63,64,65]. Legumes, in particular, are recognized as major protein contributors due to their high protein content and functional properties, with lupin seeds reaching values up to approximately 40–46% on a dry matter basis, thus significantly enhancing the nutritional profile of gluten-free formulations [66]. The superior protein content observed in TBF can be attributed to its higher proportion of millet and chickpea, as well as the intrinsic protein richness of teff compared to rice. Indeed, teff and other pseudocereals are characterized by higher levels of essential amino acids and improved protein quality compared to conventional cereals, whereas rice typically exhibits lower protein levels (approximately 7–8%) [67,68]. Additionally, chickpea and lupin inclusion provides a complementary amino acid profile, enhancing both the quantity and biological value of proteins in the final product [64,66,69].

Lipid content was also significantly higher in gluten-free formulations (2.36–3.42%) than in CDW (1.11%), reflecting the incorporation of lipid-rich ingredients such as pseudocereals, legumes, and seeds [64,68,70,71,72]. These values are consistent with the compositional ranges reported for the selected raw materials, where pseudocereals such as quinoa typically contain 4–7% lipids, legumes range between 3 and 10%, and oilseeds such as chia may reach lipid contents of approximately 30% [72].

The higher lipid content observed in TBF compared to RBF is consistent with its greater proportion of millet and chickpea, as well as the contribution of teff, which contains approximately 2–3% lipids [15]. In addition, the presence of legumes and seeds—particularly those rich in unsaturated fatty acids—further contributes to the overall lipid enrichment of the formulation. Despite this increase, lipid levels remained within technologically acceptable limits. This aspect is particularly relevant, as excessive lipid content in flour-based systems may accelerate oxidative deterioration through lipoxygenase-mediated oxidation of unsaturated fatty acids, negatively affecting flavor stability and shelf life [73]. Therefore, the lipid levels achieved in the optimized formulations represent a balanced condition, enhancing the nutritional profile without compromising product stability during storage and processing. Ash content was significantly higher in gluten-free formulations, particularly in TBF (2.19%), reflecting the higher mineral content of whole grains and less refined ingredients compared to durum wheat semolina, where bran removal reduces mineral fractions [74].

Crude fiber content, measured by the Weende method, which mainly measures insoluble fiber fractions and does not represent total dietary fiber, showed distinct differences between formulations. TBF (2.62%) exhibited values comparable to CDW (2.52%), indicating that teff-based formulations can effectively replicate the insoluble fiber profile of traditional products. This is likely due to the whole-grain nature of teff [16,75]. In contrast, RBF showed significantly lower crude fiber content (1.12%), reflecting the well-known limitation of rice-based gluten-free products [76].

Carbohydrate content was highest in RBF (71.56%) and CDW (71.01%), while TBF showed a lower value (63.64%), consistent with its higher protein, lipid, and mineral content. This shift in macronutrient composition suggests a partial replacement of starch with nutritionally dense components, which may contribute to a lower glycemic response, as previously reported for teff-based products [15].

Calorific values were slightly higher for gluten-free formulations (359.7–361.12 kcal/100 g) compared to CDW (346.55 kcal/100 g), mainly due to their increased protein and lipid content. This higher energy density may represent a nutritional advantage, particularly for individuals with celiac disease, who often experience inadequate energy and macronutrient intake [77].

Overall, the results demonstrate that the optimized gluten-free formulations, particularly TBF, offer improved nutritional quality compared to conventional durum wheat products, combining enhanced protein, lipid, and mineral contents with adequate fiber levels.

#### 3.5.2. Rheological Behavior of Liquid Batters

The rheological behavior of liquid batter plays a crucial role in determining the final quality of Algerian pancakes, particularly in relation to the development of the characteristic porous honeycomb structure. Batter viscosity directly governs gas retention and expansion during cooking, thereby influencing both density and texture.

High-viscosity systems hinder CO_2_ migration and bubble expansion, resulting in dense structures with limited aeration. Conversely, excessively low-viscosity batters lack structural integrity, leading to premature gas release and poor pore formation. As reported by Sumnu and Sahin [49], an optimal viscosity range is required to balance gas retention and expansion, ultimately maximizing product volume.

**Figure 7:**
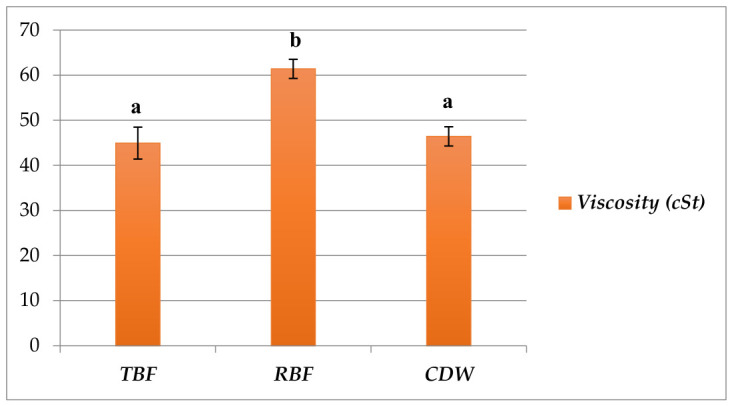
Kinematic viscosity of liquid batters from optimized gluten-free formulations (TBF and RBF) and durum wheat control (CDW). Values are expressed as mean ± standard deviation *(n* = 3). Different letters indicate significant differences according to Tukey’s post hoc test (*p* < 0.05).

The kinematic viscosity values of batters obtained from optimized gluten-free formulations and the durum wheat control are presented in Figure 7.

As shown, the rice-based formulation (RBF) exhibited the highest viscosity (61.41 ± 2.12 cSt), whereas the teff-based formulation (TBF) showed a significantly lower value (44.94 ± 3.53 cSt), statistically comparable to the control (CDW: 46.43 ± 2.21 cSt).

These differences can be attributed to formulation composition, particularly the higher seed content in RBF, which was approximately five times greater than in TBF. Seeds such as chia, flax, and garden cress are known for their mucilage-forming capacity, which leads to hydrocolloid formation upon hydration and significantly increases viscosity [24,78,79].

In addition to seed contribution, the intrinsic properties of the cereal matrix play a key role. As reported by Niba et al. [80], water-binding capacity is a primary determinant of batter viscosity, while starch swelling behavior strongly influences rheological performance. Rice-based systems are characterized by high water absorption and swelling capacity due to their small starch granule size, high surface area, and limited interaction with lipid and protein fractions [81].

These properties reduce the availability of free water in the continuous phase, thereby increasing viscosity and promoting a more structured batter system. This mechanism explains the higher viscosity observed in RBF compared to TBF and CDW.

Overall, the results confirm that batter viscosity is governed by both compositional factors (seed hydrocolloids) and intrinsic starch functionality and must be carefully controlled to achieve an optimal balance between gas retention and expansion in gluten-free pancake systems.

#### 3.5.3. Technological Characteristics of Gluten-Free Pancakes

The technological properties of pancakes obtained from optimized gluten-free formulations are presented in Table 8. Significant differences (*p* < 0.05) were observed among samples for all measured parameters, confirming the strong influence of formulation composition on structural and visual attributes.

##### Specific Volume and Density

Specific volume is widely recognized as a key indicator of the technological performance of leavened products, as it reflects the ability of the matrix to expand and retain gas during fermentation and cooking [7,82].

As shown in Table 8, both gluten-free formulations exhibited significantly higher specific volume compared to the durum wheat control (CDW). The highest value was observed for TBF (1.77 ± 0.06 cm^3^/g), followed by RBF (1.61 ± 0.01 cm^3^/g) and CDW (1.56 ± 0.03 cm^3^/g).

Density values showed an inverse trend, with TBF presenting the lowest density (0.56 ± 0.003 g/cm^3^), followed by RBF (0.62 ± 0.005 g/cm^3^) and CDW (0.64 ± 0.009 g/cm^3^). This inverse relationship confirms that lower density corresponds to greater gas incorporation and expansion capacity within the matrix.

##### Alveolar Structure

The defining characteristic of traditional Algerian pancakes is the presence of a highly developed honeycomb structure, which directly reflects batter aeration and gas retention capacity.

As reported in Table 8, TBF pancakes exhibited significantly higher porosity (977 ± 13.54) compared to both RBF (782 ± 9.61) and CDW (802 ± 32.51), which showed statistically similar values.

Conversely, alveolar size displayed an inverse trend, with TBF presenting the smallest pore diameter (0.92 ± 0.04 mm), followed by CDW (1.41 ± 0.00 mm) and RBF (1.71 ± 0.07 mm). This indicates that TBF promotes a more homogeneous and finely distributed pore structure, which is typically associated with superior textural quality.

In leavened systems, specific volume and porosity are key indicators of gas-handling efficiency, reflecting both gas retention and interfacial stability [83,84]. The results suggest a direct relationship between specific volume and porosity in the studied formulations.

The superior performance of TBF can be attributed to two main factors: (i) the higher proportion of legume proteins (chickpea), which enhances interfacial stabilization of gas cells, and (ii) the lower seed content, resulting in reduced viscosity and improved expansion capacity.

The role of seeds is particularly relevant, as their hydrocolloid properties influence batter viscosity and gas diffusion [24,85,86], while legume proteins act as natural surfactants, stabilizing the gas–liquid interface and preventing bubble coalescence [18,87,88].

Overall, the interplay between viscosity control and interfacial stabilization governs the formation of the alveolar structure in gluten-free pancake systems.

##### Color Parameters

The color parameters of gluten-free and control pancakes are reported in Table 8. Lightness (L*) values were significantly lower for both gluten-free formulations (54.76 for TBF and 56.34 for RBF) compared to CDW (66.02), indicating darker products.

This reduction in lightness can be attributed to the intrinsic pigmentation of formulation components, particularly teff in TBF and seed mixtures in RBF.

Significant differences (*p* < 0.05) were also observed for chromatic coordinates. TBF exhibited positive a* values, indicating a reddish hue associated with teff incorporation, whereas RBF and CDW showed negative a* values, corresponding to a greener tone.

For the b* parameter, RBF and CDW showed significantly higher values (31.34 and 28.76, respectively), indicating a more pronounced yellow coloration compared to TBF. This behavior is linked to the presence of naturally yellow pigments in quinoa, millet, and lupine, as well as the light color of rice flour.

Overall, the color characteristics of gluten-free pancakes are strongly influenced by the intrinsic pigments of raw materials, highlighting the importance of ingredient selection in achieving the desired visual quality.

#### 3.5.4. Antioxidant Properties of Optimized Gluten-Free Formulations

The total phenolic content (TPC), total flavonoid content (TFC), and antioxidant activities of optimized gluten-free pancakes compared with the durum wheat control (CDW) are presented in Table 9. Significant differences (*p* < 0.05) were observed among samples for most parameters, highlighting the strong influence of formulation composition on the functional profile of the final products.

TPC values ranged from 1.97 to 3.54 mg GAE/g dw, with the highest value observed for the rice-based formulation (RBF), followed by the teff-based formulation (TBF) and the control. This result indicates that the RBF matrix represents a richer source of phenolic compounds, which are known to contribute significantly to antioxidant activity and the prevention of oxidative stress-related diseases. The biological activity of these compounds is closely related to their structural characteristics [89,90,91,92].

In contrast, no significant differences were observed for total flavonoid content (TFC), which ranged from 0.12 to 0.13 mg QE/g dw across all samples. This suggests that flavonoids represent only a minor fraction (approximately 3–6.5%) of total phenolics, with non-flavonoid compounds—particularly phenolic acids—being the dominant contributors to the phenolic profile. Indeed, ferulic acid and p-coumaric acid are reported as the major phenolic acids in cereals such as rice, teff, and durum wheat, contributing to antioxidant, anti-inflammatory, and antimicrobial activities [89,90,91,92].

The total antioxidant capacity (TAC) followed the same trend as TPC, with RBF showing the highest value (4.36 mg AAE/g), confirming a strong relationship between phenolic content and overall antioxidant potential. This correlation was further supported by radical scavenging assays (ABTS and DPPH), where RBF exhibited significantly lower IC_50_ values (6.64 mg/mL for ABTS and 46.33 mg/mL for DPPH), indicating higher scavenging efficiency.

Conversely, the control sample consistently showed the lowest antioxidant performance. However, the reducing power (A_0.5_) assay revealed a different trend, with TBF reaching its effective concentration at a lower value (15.66 mg/mL) compared to RBF and CDW. This suggests that the teff-based formulation may contain specific reducing compounds (e.g., reductones or mineral-associated systems) with a higher capacity for Fe^3+^ reduction.

Overall, these results indicate that antioxidant activity is not governed solely by total phenolic concentration but also by the nature and reactivity of the bioactive compounds present.

Teff and rice are recognized as valuable gluten-free grains, rich in macro- and micronutrients as well as bioactive compounds such as phenolic acids, flavonoids, and other polyphenols. Their incorporation into food products enhances both nutritional and functional properties. In addition to their technological advantages, these cereals are suitable for individuals with celiac disease and may contribute to health benefits such as improved lipid metabolism, glycemic control, bone health, and immune function [89,90,92].

In conclusion, the substitution of durum wheat with teff or rice significantly improves the antioxidant profile of the final product. In particular, the rice-based formulation (RBF) demonstrated the highest free radical scavenging activity, while the teff-based formulation (TBF) exhibited superior reducing power, highlighting complementary functional properties between the two systems.

### 3.6. Correlation Analysis Between Nutritional, Technological and Antioxidant Parameters

The correlation matrix analysis (Table S1, Supplementary Materials) revealed significant relationships between the technological, nutritional, and functional properties of the developed pancakes.

A strong positive correlation was observed between protein content and both porosity (r = 0.959) and specific volume (r = 0.999), indicating that proteins from cereals, pseudocereals, legumes, and seeds play a crucial role in structuring the pancake matrix and enhancing its expansion capacity.

Protein content was also highly correlated with ash content (r = 0.998), reflecting the contribution of whole-grain fractions and legume-derived ingredients, which are rich in both proteins and minerals. Conversely, a strong negative correlation was found between moisture and lipid content (r = −1.000), suggesting that the incorporation of oil-rich seeds significantly reduces water retention in the final product.

Regarding antioxidant properties, total phenolic content (TPC) showed strong positive correlations with total antioxidant capacity (TAC) (r = 0.989) and reducing power (r = 0.878), confirming that phenolic compounds are the main contributors to the antioxidant potential of the formulations. In contrast, total flavonoid content (TFC) was more strongly correlated with radical scavenging activity measured by ABTS and DPPH assays (r = 0.897 and r = 0.902, respectively), suggesting a more selective involvement of flavonoids in free radical neutralization mechanisms.

As expected, density and porosity were negatively correlated (r = −0.945), confirming their inverse structural relationship. Additionally, the redness parameter (a*) showed a strong positive correlation with both protein (r = 0.993) and ash content (r = 0.983), indicating that color development is closely linked to the compositional characteristics of the raw materials.

### 3.7. Sensory Characterization of Gluten-Free and Wheat-Based Pancakes

**Figure 8:**
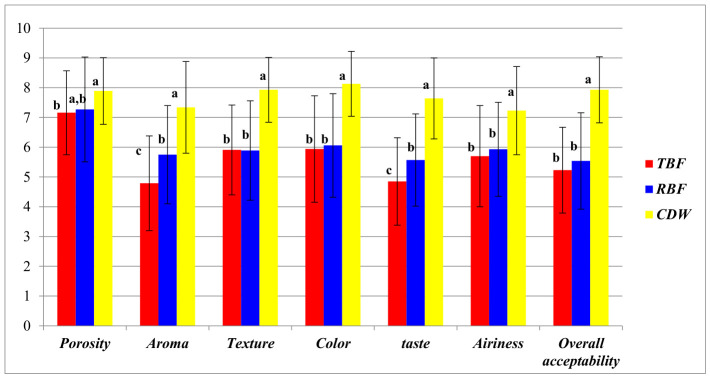
Sensory attributes of gluten-free and gluten-based pancakes. TBF: Teff-based formula, RBF: Rice-based formula, CDW: Control of durum wheat. Values are expressed as mean ± standard deviation (*n* = 65). Different letters indicate significant differences according to Tukey’s post hoc test (*p* < 0.05).

Hedonic testing was conducted to evaluate consumer acceptability of gluten-free pancakes in comparison with the durum wheat control. A nine-point hedonic scale ranging from “dislike extremely” to “like extremely”, with a neutral midpoint, was used. Panelists were asked to select the category that best represented their level of preference for each attribute. The results of the sensory characterization are presented in Figure 8.

As shown in Figure 8, significant differences (*p* < 0.05) were observed among samples for all evaluated sensory attributes, including porosity, aroma, texture, color, taste, airiness, and overall acceptability. The durum wheat control (CDW) consistently achieved the highest scores, with mean values of 7.89 for porosity, 7.34 for aroma, 7.93 for texture, 8.13 for color, 7.64 for taste, 7.23 for airiness, and 7.93 for overall acceptability.

No significant differences (*p* > 0.05) were observed between the two optimized gluten-free formulations (TBF and RBF) for porosity (7.16 vs. 7.27), texture (5.91 vs. 5.89), color (5.70 vs. 5.93), and overall acceptability (5.23 vs. 5.54). However, RBF showed significantly higher scores than TBF for aroma (5.75 vs. 4.97) and taste (5.57 vs. 4.85). This difference can be attributed to the characteristic flavor profile of teff, which may be less familiar and therefore less preferred by the panelists.

The ranking test confirmed the sensory hierarchy, positioning wheat-based pancakes first, followed by RBF and then TBF formulations. Despite lower scores compared to the control, both gluten-free formulations demonstrated satisfactory acceptability, particularly in terms of porosity, which is a key quality attribute for traditional Algerian pancakes.

These results confirm that the selected formulation strategy, based on the combination of cereals, pseudocereals, legumes, and seeds, successfully reproduced the characteristic texture and structure of traditional pancakes while maintaining acceptable sensory properties.

## 4. Conclusions

This study successfully developed gluten-free pancakes inspired by traditional Algerian formulations by combining cereals, pseudocereals, legumes, and seeds. The integration of a household survey with mixture design methodology enabled the effective translation of traditional knowledge into a scientifically optimized formulation strategy.

The optimized gluten-free pancakes (TBF and RBF) demonstrated significantly improved nutritional profiles compared to durum wheat control, particularly in terms of protein, lipid, mineral, and antioxidant content. The incorporation of teff, quinoa, and legumes contributed to enhanced bioactive compound content and antioxidant capacity, confirming the functional potential of these formulations.

From a technological perspective, the results highlighted that the combination of legume proteins and seed-derived mucilage effectively compensated for the absence of gluten, enabling the development of a stable batter structure and a characteristic porous “honeycomb” texture typical of traditional Algerian pancakes.

Sensory evaluation confirmed that, despite slightly lower scores compared to wheat-based pancakes, gluten-free formulations achieved satisfactory consumer acceptability, particularly in terms of texture and porosity. The rice-based formulation (RBF) showed a slight advantage in flavor and aroma, while the teff-based formulation (TBF) exhibited superior nutritional and functional properties.

Overall, this study demonstrates that the combination of cereals, pseudocereals, legumes, and seeds represents an effective strategy for developing nutritionally enhanced and technologically viable gluten-free products. These findings provide a valuable basis for the development of culturally adapted gluten-free foods, particularly for populations affected by celiac disease, and support future applications in the functional food and agri-food sectors.

## Figures and Tables

**Table 1:** Composition of gluten-free pancake formulations based on cereal/pseudocereal, legume, and seed components.

Formulation	Cereal/Pseudocereal Mixture	Legume Source	Seed Mixture
TBF	Teff, millet, quinoa	Chickpea	Flax, garden cress
RBF	Rice, millet, quinoa	Lupine	Chia, garden cress

TBF: Teff-based formulation; RBF: Rice-based formulation.

**Table 2:** Simplex centroid mixture design matrix for teff-based (TBF) and rice-based (RBF) gluten-free pancake formulations.

Trial	X_1_	X_2_	X_3_	TBF–Cereal/ Pseudocereal (%)	TBF–Legume (%)	TBF–Seeds (%)	RBF–Cereal/Pseudocereal (%)	RBF–Legume (%)	RBF–Seeds (%)
**1**	0.5	0.5	0	74.5	24.5	1	79.5	19.5	1
**2**	0.5	0	0.5	74.5	20	5.5	79.5	15	5.5
**3**	1	0	0	79	20	1	84	15	1
**4**	0	0	1	70	20	10	75	15	10
**5**	0	0.5	0.5	70	24.5	5.5	75	19.5	5.5
**6**	0	1	0	70	29	1	75	24	1
**7**	0.333	0.333	0.333	73	23	4	78	18	4

The design includes binary and ternary mixture combinations according to the simplex centroid scheme. X_1_: cereal/pseudocereal mixture; X_2_: legume fraction; X_3_: seed mixture. TBF: teff-based formulation; RBF: rice-based formulation.

**Table 3:** Regression coefficients and statistical parameters of linear mixture models for TBF and RBF formulations.

Formulation	Response	β_1_ (X_1_)	β_2_ (X_2_)	β_3_ (X_3_)	*p*-Value	R^2^	R^2^adj
**TBF**	Specific volume (cm^3^/g)	−0.0025	−0.1186	0.409	<0.05	0.9982	0.9894
	Lightness (L*)	1.31	7.06	21.43	<0.05	0.9963	0.9776
	Porosity (%)	−26.60	−130.7	−1153	<0.05	0.9904	0.9422
	Viscosity (cSt)	−1.97	−17.04	−41.19	<0.05	0.9941	0.9647
**RBF**	Specific volume (cm^3^/g)	0.019	0.077	0.46	>0.05	0.7954	0.7954
	Lightness (L*)	−0.03	−8.13	35.96	<0.05	0.9904	0.9423
	Porosity (%)	19.23	28.67	−765.2	<0.05	0.9840	0.9037
	Viscosity (cSt)	−0.95	−16.31	39.18	<0.05	0.9619	0.7713

TBF: teff-based formulation; RBF: rice-based formulation. X_1_: cereal/pseudocereal mixture; X_2_: legume fraction; X_3_: seed mixture. β_1_, β_2_, β_3_: linear regression coefficients of mixture components. R^2^: coefficient of determination; R ^2^adj: adjusted coefficient of determination. Significant effects were considered at *p* < 0.0.

**Table 4:** Technological properties of pancakes and rheological behavior of batter for teff-based formulations (TBF) according to the mixture design.

Trial	X_1_ (%)	X_2_ (%)	X_3_ (%)	Specific Volume (cm^3^/g)	Porosity	Lightness (L*)	Viscosity (cSt)
**F1**	74.5	24.5	1	1.78 ± 0.01 ^a^	936.00 ± 63.64 ^ab^	50.50 ± 4.65 ^abc^	43.66 ± 2.83 ^c^
**F2**	74.5	20	5.5	1.43 ± 0.21 ^bc^	925.67 ± 169.00 ^b^	40.96 ± 6.22 ^d^	45.15 ± 2.12 ^c^
**F3**	79	20	1	1.67 ± 0.03 ^ab^	795.00 ± 97.00 ^b^	52.14 ± 3.45 ^ab^	30.60 ± 3.45 ^d^
**F4**	70	20	10	1.35 ± 0.01 ^c^	246.50 ± 11.50 ^c^	44.72 ± 3.91 ^cd^	42.59 ± 0.70 ^c^
**F5**	70	24.5	5.5	1.48 ± 0.03 ^bc^	224.50 ± 45.50 ^c^	50.32 ± 1.30 ^abc^	58.64 ± 4.24 ^a^
**F6**	70	29	1	1.77 ± 0.02 ^a^	1010.00 ± 2.00 ^a^	54.26 ± 4.25 ^a^	44.94 ± 7.07 ^c^
**F7**	73	23	4	1.53 ± 0.00 ^abc^	800.50 ± 176.50 ^b^	47.10 ± 4.22 ^bcd^	54.57 ± 3.54 ^b^

TBF: teff-based formulation. X_1_: cereal/pseudocereal mixture; X_2_: legume fraction; X_3_: seed mixture. Values are expressed as mean ± standard deviation (*n* = 3). Means followed by different superscript letters within the same column are significantly different according to Tukey’s post hoc test (*p* < 0.05).

**Table 5:** Technological properties of pancakes and rheological behavior of batter for rice-based formulations (RBF) according to the mixture design.

Trial	X_1_ (%)	X_2_ (%)	X_3_ (%)	Specific Volume (cm^3^/g)	Porosity	Lightness (L*)	Viscosity (cSt)
**F1**	79.5	19.5	1	1.39 ± 0.01 ^b^	525.50 ± 79.50 ^b^	57.16 ± 7.83 ^ab^	62.49 ± 5.66 ^a^
**F2**	79.5	15	5.5	1.53 ± 0.10 ^ab^	713.00 ± 19.00 ^a^	38.65 ± 0.35 ^c^	45.58 ± 10.00 ^b^
**F3**	84	15	1	1.41 ± 0.08 ^b^	760.50 ± 103.50 ^a^	50.28 ± 6.26 ^abc^	46.86 ± 6.36 ^b^
**F4**	75	15	10	1.44 ± 0.10 ^ab^	451.50 ± 19.00 ^bc^	48.56 ± 10.20 ^bc^	65.91 ± 2.83 ^a^
**F5**	75	19.5	5.5	1.63 ± 0.07 ^a^	744.50 ± 1.50 ^a^	56.90 ± 4.40 ^ab^	64.20 ± 1.40 ^a^
**F6**	75	24	1	1.38 ± 0.17 ^b^	377.50 ± 9.50 ^c^	59.80 ± 6.00 ^a^	70.19 ± 8.49 ^a^
**F7**	78	18	4	1.50 ± 0.07 ^ab^	770.00 ± 47.00 ^a^	52.43 ± 4.11 ^ab^	62.06 ± 4.24 ^a^

RBF: rice-based formulation. X_1_: cereal/pseudocereal mixture; X_2_: legume fraction; X_3_: seed mixture. Values are expressed as mean ± standard deviation (*n* = 3). Means followed by different superscript letters within the same column are significantly different according to Tukey’s post hoc test (*p* < 0.05).

**Table 6:** Predicted and experimental values and desirability indices of optimized TBF and RBF gluten-free pancake formulations.

Samples	Quality Parameters	Predicted Values	Observed Values	Individual Desirability	Composite Desirability
TBF (X_1_ = 70.1%, X_2_ = 28.90%, X_3_ = 1%)	Specific volume	1.77 ^a^	1.77 ± 006 ^a^	0.99	D = 0.989
	Porosity	1003 ^a^	977 ± 3.45 ^a^	0.98	
	Lightness	54.08 ^a^	54.76 ± 2.6 ^a^	0.98	
	viscosity	44.91 ^a^	44.94 ± 3.53 ^a^	0.99	
RBF (X_1_ = 75.54%, X_2_ = 18.99%, X_3_ = 5.47%)	Specific volume	1.60 ^a^	1.61 ± 0.01 ^a^	0.88	D = 0.91
	Porosity	756.05 ^a^	782.33 ± 9.61 ^a^	0.80	
	Lightness	55.66 ^a^	56.34 ± 0.34 ^a^	0.96	
	Viscosity	64.20 ^a^	61.41 ± 2.12 ^a^	0.99	

TBF: teff-based formulation; RBF: rice-based formulation. X_1_: cereal/pseudocereal mixture; X_2_: legume fraction; X_3_: seed mixture. Values are expressed as mean ± standard deviation (*n* = 3). Means followed by the same superscript letter within each parameter indicate no significant difference between predicted and observed values according to Tukey’s post hoc test (*p* > 0.05). D: composite desirability index.

**Table 7:** Proximate composition and calorific value of optimized gluten-free pancakes compared with durum wheat control.

Sample	Moisture (%)	Proteins (%)	Lipids (%)	Ash (%)	Fiber (%)	Carbohydrates (%)	Energy (kcal/100 g)
TBF	9.54 ± 0.05 ^c^	18.59 ± 0.10 ^a^	3.42 ± 0.06 ^a^	2.19 ± 0.15 ^a^	2.62 ± 0.13 ^a^	63.64	359.70
RBF	10.25 ± 0.09 ^b^	13.41 ± 0.08 ^b^	2.36 ± 0.06 ^b^	1.30 ± 0.03 ^b^	1.12 ± 0.02 ^b^	71.56	361.12
CDW	11.07 ± 0.33 ^a^	12.08 ± 0.56 ^c^	1.11 ± 0.16 ^c^	1.16 ± 0.07 ^b^	2.52 ± 0.01 ^a^	71.01	346.55

TBF: teff-based formulation; RBF: rice-based formulation; CDW: control durum wheat. Values are expressed as mean ± standard deviation *(n* = 3). Carbohydrates were calculated by difference. Energy values were calculated using Atwater factors (4 kcal/g for proteins and carbohydrates, 9 kcal/g for lipids). Means followed by different superscript letters within the same column are significantly different according to Tukey’s post hoc test (*p* < 0.05).

**Table 8:** Technological properties, alveolar structure, and color parameters of optimized gluten-free pancakes compared with durum wheat control.

Sample	Specific volume (cm^3^/g)	Density (g/cm^3^)	Porosity	Alveolar size (mm)	L*	a*	b*
**TBF**	1.77 ± 0.06 ^a^	0.56 ± 0.003 ^c^	977 ± 13.54 ^a^	0.92 ± 0.04 ^c^	54.76 ± 2.60 ^b^	2.24 ± 0.71 ^a^	25.14 ± 1.97 ^a^
**RBF**	1.61 ± 0.01 ^b^	0.62 ± 0.005 ^b^	782 ± 9.61 ^b^	1.71 ± 0.07 ^a^	56.34 ± 0.34 ^b^	−2.82 ± 0.84 ^b^	31.34 ± 2.07 ^b^
**CDW**	1.56 ± 0.03 ^c^	0.64 ± 0.009 ^a^	802 ± 32.51 ^b^	1.41 ± 0.00 ^b^	66.02 ± 1.27 ^a^	−5.14 ± 1.27 ^c^	28.76 ± 3.77 ^ab^

TBF: teff-based formulation; RBF: rice-based formulation; CDW: control durum wheat. Values are expressed as mean ± standard deviation (*n* = 3). Means followed by different superscript letters within the same column are significantly different according to Tukey’s post hoc test (*p* < 0.05).

**Table 9:** Antioxidant properties of optimized gluten-free pancakes compared with durum wheat control.

Sample	TPC (mg GAE/g dw)	TFC (mg QE/g dw)	TAC (mg AAE/g dw)	ABTS IC_50_ (mg dw/mL)	DPPH IC_50_ (mg dw/mL)	Reducing Power A_0_._5_ (mg dw/mL)
TBF	2.48 ± 0.61^b^	0.12 ± 0.02^a^	3.19 ± 0.02^b^	11.18 ± 0.44^b^	62.10 ± 0.48^b^	15.66 ± 0.48^c^
RBF	3.54 ± 0.01^a^	0.12 ± 0.01^a^	4.36 ± 0.09^a^	6.64 ± 0.34^c^	46.33 ± 0.25^c^	22.91 ± 0.11^a^
CDW	1.97 ± 0.02^c^	0.13 ± 0.01^a^	2.20 ± 0.01^c^	16.89 ± 0.77^a^	82.78 ± 5.88^a^	17.00 ± 0.32^b^

TBF: teff-based formulation; RBF: rice-based formulation; CDW: control durum wheat. TPC: total phenolic content (mg GAE/g dw); TFC: total flavonoid content (mg QE/g dw); TAC: total antioxidant capacity (mg AAE/g dw). Values are expressed as mean ± standard deviation (*n* = 3). IC_50_: concentration required to inhibit 50% of radical activity. Means followed by different superscript letters within the same column are significantly different according to Tukey’s post hoc test (*p* < 0.05).

## Data Availability

The original contributions presented in this study are included in the article/Supplementary Materials. Further inquiries can be directed to the corresponding authors.

## Supplementary Materials

The following supporting information can be downloaded at: https://www.mdpi.com/article/10.3390/foods15111867/s1, Figure S1. Flow diagram for the preparation of gluten-free Algerian pancakes. Table S1. Nutritional, Technological and Physicochemical Correlation.

## Abbreviations

The following abbreviations are used in this manuscript:

RBF	Rice-Based Formulation
TBF	Teff-Based Formulation
TAC	Total Antioxidant Capacity
TFC	Total Flavonoid Content
TPC	Total Phenolic Content
