# Supplementary material for: Development and Optimization of Rice and Teff Based Gluten-Free Mixes for Traditional Algerian Pancakes: Evaluation of Technological Properties, Nutritional Quality, and Sensory Attributes
Source: Foods. 2026 May 25;15(11):1867. doi: 10.3390/foods15111867 (PMC13257189; doi:10.3390/foods15111867)
Supplement: Supplementary file 1 [file foods-15-01867-s001.zip › foods-4302180-supplementary.pdf]

Supplementary Material

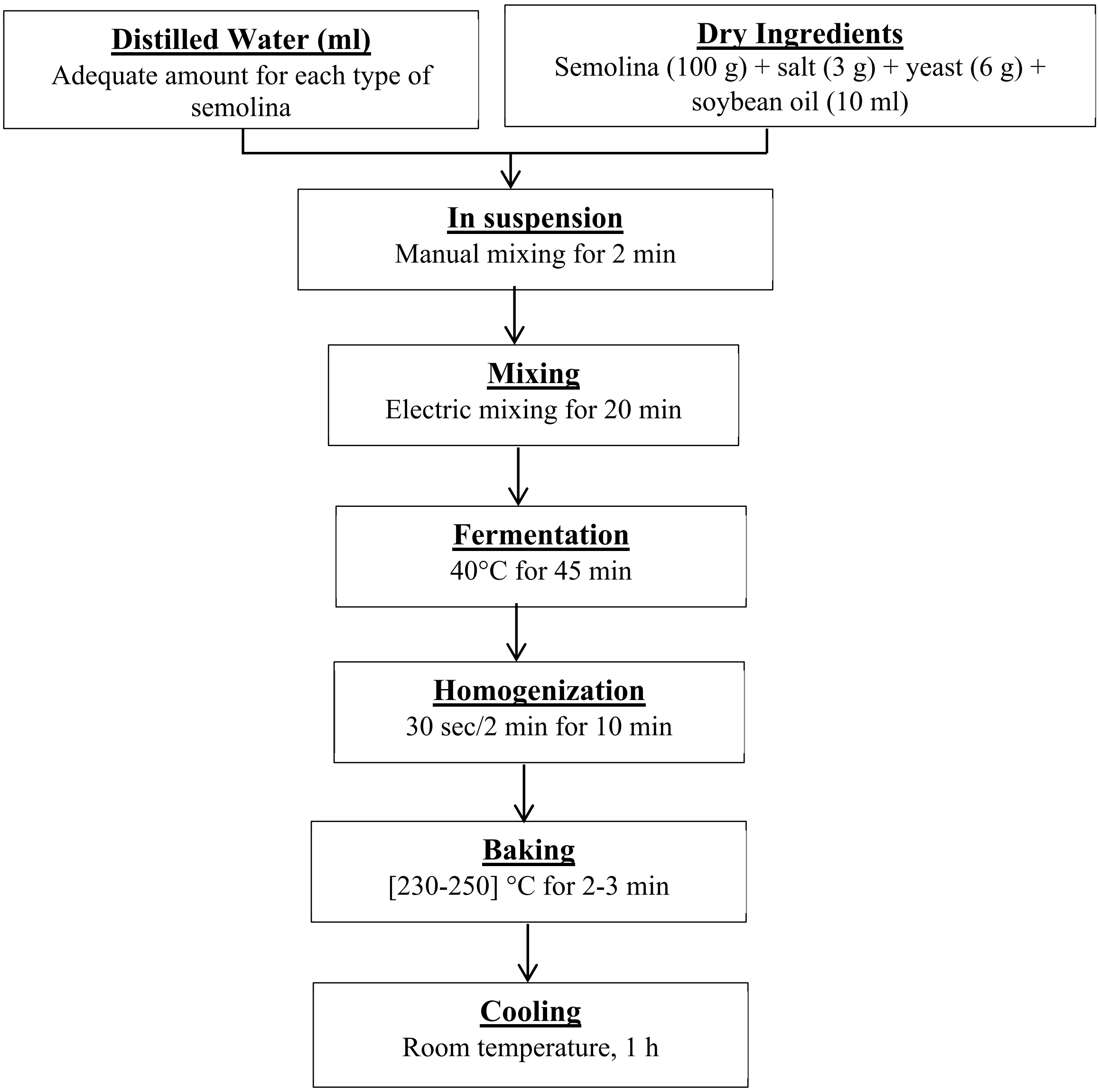

Figure S1. Flow diagram for the preparation of gluten-free Algerian pancakes.

Table S1. Nutritional, Technological and Physicochemical Correlation.

Variables	Moisture	Proteins	Lipids	Ash	Fibers	CRBT	TPC	TFC	TAC	ABTS	DPPH	RED	Viscosity	SV	Density	Porosity	Average size	L*	a*	b*
Moisture	**1**																			
Proteins	**-0,932**	**1**																		
Lipids	**-1,000**	**0,930**	**1**																	
Ash	**-0,905**	**0,998**	**0,902**	**1**																
Fibers	-0,018	0,379	0,012	0,442	**1**															
CRBT	**0,810**	**-0,967**	**-0,806**	**-0,982**	-0,602	**1**														
TPC	-0,357	-0,005	0,363	-0,074	**-0,927**	0,259	**1**													
TFC	**0,886**	-0,658	**-0,889**	-0,605	0,447	0,445	**-0,750**	**1**												
TAC	-0,494	0,146	0,499	0,077	**-0,860**	0,110	**0,989**	**-0,841**	**1**											
ABTS	0,590	-0,257	-0,595	-0,190	**0,797**	0,004	**-0,965**	**0,897**	**-0,994**	**1**										
DPPH	0,599	-0,269	-0,604	-0,202	**0,789**	0,015	**-0,962**	**0,902**	**-0,992**	**1,000**	**1**									
RED	0,133	-0,483	-0,127	-0,542	**-0,993**	0,689	**0,878**	-0,342	**0,796**	**-0,722**	**-0,714**	**1**								
Viscosity	0,040	-0,399	-0,034	-0,462	**-1,000**	0,619	**0,919**	-0,427	**0,849**	**-0,783**	**-0,776**	**0,996**	**1**							
SV	**-0,944**	**0,999**	**0,942**	**0,995**	0,346	**-0,958**	0,030	-0,684	0,181	-0,291	-0,303	-0,451	-0,367	**1**						
Density	**0,948**	**-0,999**	**-0,947**	**-0,993**	-0,334	**0,954**	-0,043	0,693	-0,193	0,304	0,315	0,440	0,355	**-1,000**	**1**					
Porosity	**-0,792**	**0,959**	**0,788**	**0,976**	0,625	**-1,000**	-0,287	-0,418	-0,140	0,026	0,014	-0,710	-0,642	**0,949**	**-0,945**	**1**				
Average size	0,581	**-0,836**	-0,576	**-0,872**	**-0,824**	**0,948**	0,552	0,138	0,420	-0,314	-0,303	**0,884**	**0,837**	**-0,816**	**0,809**	**-0,957**	**1**			
L*	**0,938**	**-0,750**	**-0,940**	**-0,702**	0,329	0,557	-0,658	**0,992**	**-0,764**	**0,833**	**0,839**	-0,218	-0,307	**-0,772**	**0,780**	-0,532	0,264	**1**		
a*	**-0,968**	**0,993**	**0,967**	**0,983**	0,268	**-0,931**	0,113	**-0,742**	0,261	-0,369	-0,380	-0,376	-0,289	**0,997**	**-0,998**	**0,919**	**-0,766**	**-0,822**	**1**	
b*	0,547	**-0,813**	-0,542	**-0,851**	**-0,847**	**0,934**	0,586	0,096	0,457	-0,353	-0,342	**0,902**	**0,859**	**-0,792**	**0,784**	**-0,944**	**0,999**	0,224	**-0,739**	**1**
